# Dynamics of soil organic carbon of jhum agriculture land-use system in the heterogeneous hill of Arunachal Pradesh, India

**DOI:** 10.1038/s41598-023-38421-1

**Published:** 2023-07-27

**Authors:** Jitendra Kumar, H. Kalita, Wangnem Rekhung, Rajesh A. Alone, Thejangulie Angami, Doni Jini, Badapmain Makdoh, Letngam Touthang, Nirmal Khatri, A. P. Singh, Nishant K. Sinha, Dhiraj Kumar, R. S. Chaudhary

**Affiliations:** 1 ICAR- Indian Institute of Soil Science, Nabibagh, Berasia Road, Bhopal, Madhya Pradesh India; 2 ICAR RC for NEH Region, AP Centre, Basar, Arunachal Pradesh India; 3 ICAR-Indian Institute of Farming Systems Research, Modipuram, Meerut, Uttar Pradesh India; 4 SAS Nagaland University, Medziphema Campus, Kohima, Nagaland India

**Keywords:** Microbiology, Plant sciences, Biogeochemistry, Climate sciences, Environmental sciences, Solid Earth sciences

## Abstract

Land-use conversion affects soil organic carbon (SOC) dynamics. Therefore, an in-depth study of change in SOC, SOC pool, fractions of SOC and enzymatic activities of soil microbial biomass carbon (SMBC) and dehydrogenase (DHA) with the conversion of forest land to *jhum*, fallow *jhum* and settle cultivation use has been undertaken on the hills of Arunachal Pradesh of India. Geo-referenced soil samples from eight different locations, each from different land uses were collected at three depth. One part of the soil sample had been used for the analysis of SOC and its carbon fraction. The second portion was kept in a deep freezer for determining SMBC and DHA. The third part was used for the analysis of bulk density. The result revealed that the highest loss of SOC pool was recorded in *jhum* land (41.8 to 13.4%), and the labile carbon was also found to decrease in *jhum* land. The highest SMBC was observed on the surface soil of the natural forest; the highest DHA was found in the natural forest; and the lowest DHA was recorded in *jhum* land. This study found that the converting natural forest to *jhum* reduces SOC storage, enzymatic activities and C fractions significantly whereas fallow *jhum* shows sign of recovery because all of these parameters improved when compared to the *jhum* land-use system. This study also confirms that the fallow period helps restore the initial situation.

## Introduction

Arunachal Pradesh is situated in the northeast Himalayan region of India, where *jhum* cultivation (JC) is one of the main sustenance activities of small-scale farmers and rural populations for food requirements, economic growth, and cultural activities.To carry out the *jhum* cultivation, farmers are going for deforestation and land use transformation, which are vital ecological issues in these regions. The transformation of natural forests into *jhum* significantly modifies the soil properties and their processes, and therefore, soil functioning^1,2^. This has also been reported in earlier studies that land use and cover alter the soil's organic carbon dynamics due to the influence of physical, chemical, and biological properties, which eventually modify the soil fertility and quality^3^. Further, the variability in land cover also impacts the soil microbial activities, which influence the soil organic carbon pool considerably. This is due to changes in the rate of input (e.g. addition of plant litter) and rate of output (e.g. mineralization of soil organic carbon) of soil organic matter (SOM) and land management operations^2,4^.

The forests influence the concentration of carbon present in the atmosphere^5^. As a result transformation of natural forests into other land uses such as *jhum*, plantations and crop lands for socioeconomic activities results in the modification of soil properties and its processes. A study in the northern part of India has reported that continuous cultivation resulted in a loss of 21–36% of total organic carbon as compared to uncultivated soils^6^, which is comparatively less than the values (30–60%) SOC loss reported in other agro-climatic regions of India^7^. When natural forests are converted to different land uses, the soil as a whole gets disrupted, leading to an enhancement in the rate of mineralization of organic matter by microbial activities and subsequently leading to SOC loss^8^. A wide range of SOC concentrations from 0.85 to 3.56%^9^ with SOC stock of 20–40 Mg/ha^–^ in the top 0–30 cm of soil depth^10^ has also been reported in Arunachal Pradesh. The SOC in Banana cultivation was 1.75%^11^ as reported in North Eastern Himalayan Region.

Deforestation and the shift in vegetation composition are responsible to altering soil structure and physicochemical properties^12,13^. It is supposed that these processes also affects soil microbial and enzymatic activities and soil nutrient cycling^14^. This study hypothesizes that transformation in land uses alter the soil microbiological and enzyme activities, and therefore, soil carbon and associated attributes could be used as sensitive indicators of soil health. The objective of this investigation is to understand the effect of different land-use types and deforestation on soil organic carbon (SOC pool) and different fractions of carbon. Further, this study will also determine the activity of selected soil enzymes involved in the cycling of SOC, i.e. SMBC and Dehydrogenase under selected land use and cover types.

## Materials and methods

### Description of the study area

**Figure 1:**
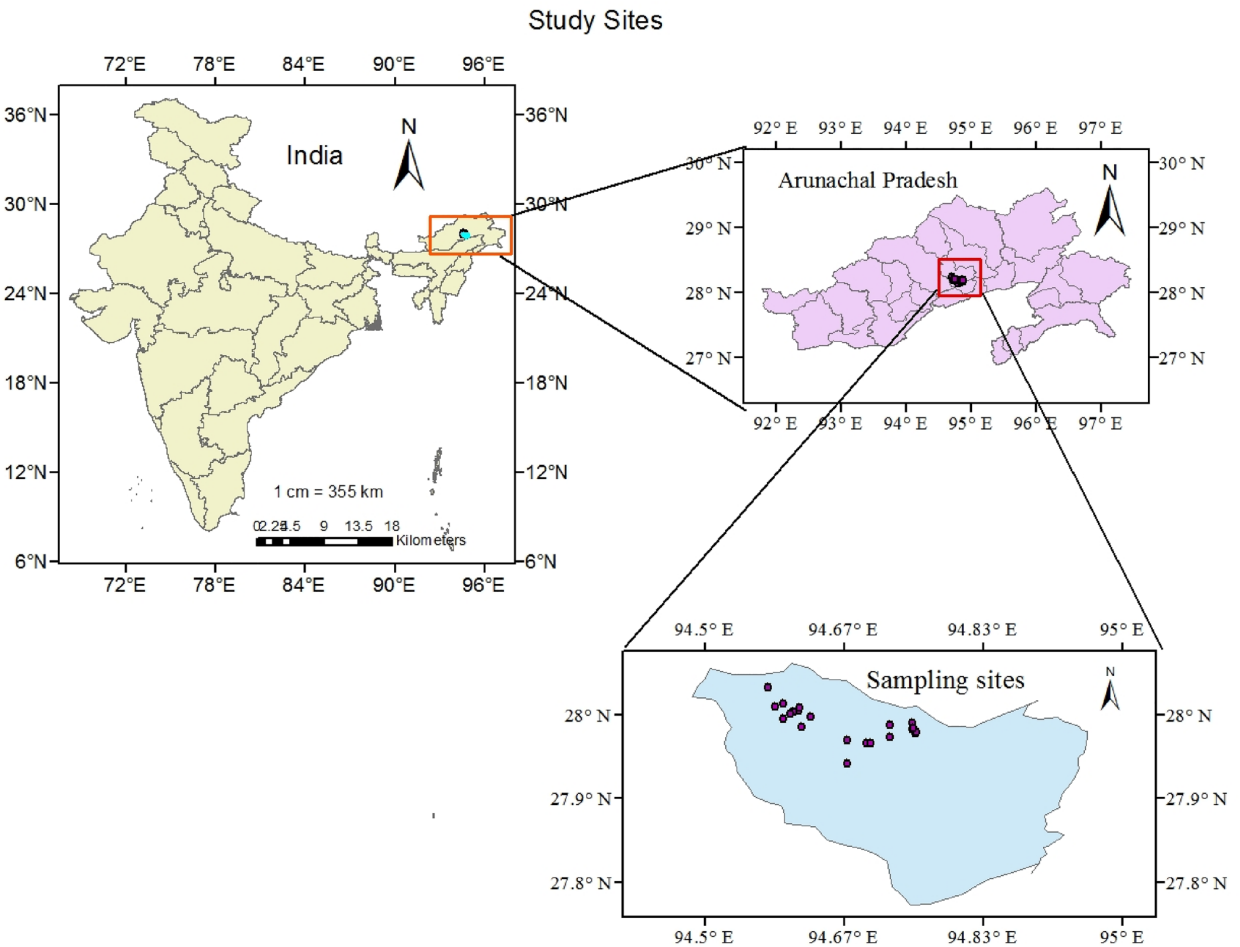
Study site: Arunachal Pradesh Northeast Himalayan region of India, This map was prepared by the first author and co-author irmal Khatri Bapapain Makdoh and Letngam Touthang with the help of ArcGIS 10.4 (http://www.esri.com/software/arcgis) and does not require any permission from anywhere.

The research has been conducted at ICAR RC for NEH Region, Arunachal Pradesh Centre, Basar, in district West Siang now Leparada (Fig. 1), which is situated between longitude 93.57°E to 95.23°E and latitude 27.69°N to 29.20°N. It has a land area of 7643 square kilometersand borders Upper and East Siang in the east, China towards the north, and Upper Subansiri at West, and Assam in the South. The climatic state of the district varies from tropical to sub-tropical due to the undulating terrain. The topography of the West Siang region has an average elevation of 578 m above mean sea level, and the mean annual precipitation is approximately 2100 mm. Due to adequate rainfall the soil moisture availability for the crops generally lasts more than 270 days in a year. A detailed description of the different land uses selected is provided in Table 1.

**Table 1:** Background information of the study sites.

Land use system	Altitude (m)	Latitude	Longitude	Soil texture	Soil order	Management	History of the cultivation
Jhum	646	27.9664	94.6931	Silty clay	Entisol	Mixed cropping like vegatables, Pulses, Maize etc	Jhum’s current year
Natural forest	647	27.9661	94.6978	Sandy Clay	Entisol	No Management	The unknown history of a naturally grown forest
Falow jhum	711	27.5703	94.6708	Sandy Clay	Entisol	Allow to regenerate the vegetation	Fallow from the last 2 years after a 2-year jhum
Pine apple	743	27.9422	94.7006	Sandy clay	Entisol	Occasionally, kitchen and poultry waste are added	n the last 7 years, Jhum land has settled for pineapple cultivation

### Soil sampling, processing, and analysis

Geo-referenced The soil samples from georeferenced locations has been collected at the depth of (0–15, 15–30, and 30–45 cm depth) from the core sampler for bulk density and from the auger sampler for other parameters. The sample is collected from 8 different locations and each location has four land uses: *jhum*, natural forest, fallow *jhum,* and Pineapple plantation, Each location is divided on a 50 × 50 m grid, and each grid divided into three parts. Thus in total, 288 composite samples (4 land uses × 8 sampling locations × 3 depths × 3 replications) were collected. A portion of the soil sample was air-dried, ground, and sieved through a 2.00-mm sieve and then used for the analysis of chemical properties such as soil pH, soil organic carbon (SOC), and carbon fraction. The soil organic carbon was determine by wet digestion method^15^. The second part was passed through 0.5-sieve and kept in a deep freezer for determination of biological properties: soil microbial carbon biomass (SMBC) and soil dehydrogenase activity (DHA). The third part was used for the analysis of physical properties such as bulk density.

### Analysis of soil carbon fractions and pools

Soil organic carbon fractions (SOC) were determined under an increasing grade of the oxidising condition by employing three aqueous sulfuric acid concentration solutions in the ratios of 0.5:1, 1:1, and 2:1, respectively^16^. Thus the estimated carbon content leads to the partitioning of SOC into the following four different organic carbon fractions in decreasing order of oxidisability. Fraction I (very labile): organic carbon oxidisable under 0.5:1 H_2_SO_4_, Fraction II (labile): The difference in carbon oxidisable under 1:1 and 0.5:1 H_2_SO_4_, Fraction III (less labile): The difference in carbon oxidisable under 2:1 and 1:1 H_2_SO_4_, Fraction IV (non-labile): The difference between soil organic carbon and carbon oxidisable under 2:1 H_2_SO_4_. The active pool of organic carbon was calculated by adding fraction I and fraction IIwhile the passive pool of organic carbon is determined by the adding of fraction III and fraction IV. An active pool of organic carbon represents the amount of organic carbon present in an readily oxidisable form. While the passive pool of organic carbon represents the carbon fraction resistant to decomposition.

The soil organic carbon (SOC) pool (Mg C ha^−1^) was determined by the following equation:

$${\text{SOC pool }}\left( {{\text{Mg C ha}}^{{ - {1}}} } \right)\, = \,\left[ {{\text{SOC }}\left( \% \right)\, \times \,\rho {\text{b }}\left( {{\text{Mg m}}^{{ - {3}}} } \right)\, \times \,{\text{Depth }}\left( {\text{m}} \right)\, \times \,{1}0{\text{4 m}}^{{2}} {\text{ha}}^{{ - {1}}} } \right]\, \div \,{1}00.$$

### Analysis of soil biological properties

The dehydrogenase activity was determined by reducing 2, 3, 5-triphenyl tetrazolium chloride^17^. A standard curve of triphenylformazan (TPF) in methanol was used to determination of the DHA activity, and it is expressed in terms of μg TPFg^−1^ soil h^−1^.For the determination of Soil Microbial biomass carbon the chloroform fumigation-extraction method^18^ was used and it is expressed in μg g^–1^ dry soil.

### Statistical analysis

For statistical analysis of data regarding the different properties of soils in our study, a general linear model of SAS 9.4 (SAS Institute, 2003) was utilized. The least significant difference between LSD (p = 0.05) was computed to compare means among various land-use systems.

## Results

### Soil organic carbon content, bulk density and Soil pH

The result of the present study revealed that different land-use systems have an significant influence on soil pH and bulk density. Soil pH ranged from 4.8 in fallow *jhum* to 5.4 in natural forests. In general, they were showing an increasing trend of pH deeper soil layer i.e. with the increasing soil depth. The highest pH was recorded at 30–45 cm soil layers of respective land-use systems (Tables 2 and 3). The lowest values of pH were recorded at 0–15 cm, and the highest was recorded at 30–45 cm depth. Moreover, the soil layer of 30–45 cm does not show a significant effect of land use on soil pH.

**Table 2:** The impact of land use change on pH and bulk density.

Land use system	pH			BD (Mgm^–3^)		
	0–15 cm	15–30 cm	30–45 cm	0–15 cm	15–30 cm	30–45 cm
Jhum	4.8^b^	4.9^b^	5.17	1.29^a^	1.27^a^	1.29^a^
Natural forest	5.3^a^	5.4^a^	5.22	1.09^d^	1.13^c^	1.13^c^
Fallow jhum	4.8^b^	4.9^b^	5.3	1.24^b^	1.23^b^	1.25^b^
Pine apple	5.2^a^	5.2^a^	5.35	1.13^c^	1.17^b^	1.20^b^

Means followed by different letters (a–d) are significantly different from the LSD (least significant difference) values at p = 0.05. Significant differences shown are in land uses.

**Table 3:** Effect of change in land use on soil organic carbon and soil organic pool.

Land use system	SOC (%)			SOC Pool (Mg ha^−1^)		
	0–15 cm	15–30 cm	30–45 cm	0–15 cm	15–30 cm	30–45 cm
Jhum	1.19^c^	1.05^d^	0.95^b^	23.13^c^	19.95^c^	18.39^b^
Natural forest	2.43^a^	1.75^a^	1.25^a^	39.73^a^	29.53^a^	21.24^a^
Fallow jhum	1.24^c^	1.14^c^	0.98^b^	24.25^c^	21.03^c^	18.32^b^
Pine apple	1.68^b^	1.39^b^	0.99^b^	29.23^b^	24.4^b^	17.72^b^

Means followed by different letters (a–d) are significantly different from the LSD (least significant difference) values at p = 0.05. Significant differences shown are in land uses.

The bulk density is significantly influenced by the land use system across all soil layers. The lowest bulk density values (1.09 Mgm^–3^) were found in natural forests 0–15 cm soil layers, while the highest (1.29 Mgm^–3^) were found in *jhum* land uses 0–15 and 30–45 cm soil layers. The natural forest and pineapple land uses showed an increasing trend in bulk density across the soil depth. However, in *jhum* and fallow *jhum* land, the soil layer (15–30 cm) recorded a relatively lower bulk density than the upper layer. The soil organic carbon has also shown significant changes under different land-use systems. It is gradually decreasing with increasing soil depth from 0 to 45 cm, and the highest SOC content was recorded on surface soil i.e. 0–15 cm depth irrespective of land use system (Table 3). The natural forest had the highest SOC (2.43% in 0–15 cm, 1.75 in 15–30 cm and 1.25% in 30–45 cm depth), followed by the pineapple-based land-use system (1.68% in 0–15 cm, 1.39% in 15–30 cm, and 0.99% 30–45 cm soil layer), fallow jhum (1.24% in 0–15 cm, 1.4% in 15–30 cm, and 0.98% 30–45 cm soil layer) and lowest was recorded in *jhum* (1.19% in 0–15 cm, 1.05% in 15–30 cm, and 0.95% 30–45 cm soil layer). When compared to the natural forest at surface soil, jhum land had the greatest depletion in SOC content at all depths. Up to 15 cm soil layer 50.8%, from 15-30 cm soil layer 39.8%, and from 30–45 cm soil layer 23.9% depletion was recorded. Continuous cultivation of 2–3 crops prior to leaving land fallow resulted in reduction and further showed signs of recovery as SOC depletion was less than that of jhum land and natural forest. A minimum loss in SOC of 30% at 0–15 cm, 30% at 15–30 cm, and 21% at 30–45 cm was observed with the cultivation of pineapples.

### Effects of land use transformation on carbon pools

Land use transformation has been found a significant effect on the SOC pools across all soil depths (Table 3). The SOC storage ranged from 39.73 Mg ha^−1^ in natural forests to 23.13 Mg ha^−1^ in *jhum* land use. The natural forest has a significantly higher SOC pool across all soil layers, followed by the Pineapple land use, fallow *jhum,* and the *jhum* land-use system, which has the lowest. . At 0–15 and 15–30 cm depth, the *jhum* and fallow *jhum* were significantly at par with each other, while at 30–45 cm soil layer, except for the natural forest, all three land-use systems at par with each other, whereas the natural forest recorded significantly higher SOC pool. In this study, it is revealed that the conversion of natural forest land to different land-use systems depleted the SOC stock. Jhum land had highest depletion of SOC pool (41.8% in 0–15 cm, 32.4% in 15–30 cm and 13.4% 30–45 cm soil layer), followed by fallow *jhum* (38.9% in 0–15 cm, 28.7% in 15–30 cm and 13.7% 30–45 cm soil layer), and pineapple had the lowest depletion (26.4% in 0–15 cm, 17.3% in 15–30 cm and 16.6% 30–45 cm soil layer). The maximum depletion of soil organic carbon pool (ranging from 41.7 to 26.4%has been observed on the surface soil layer (0–15 cm), and the loss is gradually decreased with the soil depth; the lowest depletion was found on the 30–45 cm soil layer, ranging from 16.6 to 13.4%.

### Carbon fractions are influenced by the land use system

A significant effect was observed in soil carbon fractions among the various land uses in all soil layers (Table 4). The natural forest had the highest very labile carbon (9.53 g Kg^–1^ soil) followed by the pineapple (3.68 g Kg^–1^ soil) and the lowest was found in *jhum* land use. Moreover, at the 0–15 cm soil layer, except for natural forests, all other land uses were statistically on par with each other. A similar trend was also found for labile carbon. The non-labile carbon of the pineapple land use was found to be statistically at par with that of natural forest. This suggests that when natural forest land is converted into *jhum* or horticulture based land-use systems, such as pineapple land use, the very labile and labile carbon start depleting, while, the non-labile carbon remain preserved. The very labile carbon, labile carbon, less labile and non-labile ranged from 2.84 to 9.64 g Kg^–1^ soil, 1.44 g to 3.67 g Kg^–1^ soil, 1.05 to 2.70 g Kg^–1^ soil and 5.02 to 9.06 g Kg^–1^ soil respectively, at 0–15 cm soil layer.

**Table 4:** Effect of land use change in on different soil organic fractions (Mg ha^−1^).

Land use	Very labile	Labile	Less labile	Non labile	Active pool	Passive pool
0–15 cm						
Jhum	2.84^b^	1.44^b^	2.70^a^	5.02^b^	4.27^c^	7.72^b^
Natural forest	9.53^a^	3.67^a^	2.01^a^	9.06^a^	13.21^a^	11.06^a^
Fallow jhum	2.86^b^	1.76^b^	1.44^b^	6.28^b^	4.62^c^	7.71^b^
Pine aple	3.68^b^	2.58^ab^	1.05^b^	9.02^a^	6.26^b^	10.07^a^
15–30 cm						
Jhum	2^c^	1.36^d^	3.16	3.98	3.36^d^	7.14^b^
Natural forest	5.33^a^	5.01^a^	2.64	4.48	10.34^a^	7.13^b^
Fallow jhum	2.33^b^	1.8^c^	1.31	5.95	4.14^d^	7.26^b^
Pine aple	2.33^b^	3.36^b^	1.34	6.90	5.69^b^	8.24^a^
30–45 cm						
Jhum	1.67^b^	1.61^b^	6.52^b^	3.01	3.27^b^	6.26
Natural forest	1.11^b^	4.44^a^	7.75^a^	4.78	5.55^a^	6.98
Fallow jhum	2.33^a^	1.37^b^	5.45^b^	4.35	3.71^b^	6.09
Pine aple	2.33^a^	2.58^a^	7.03^a^	2.87	4.91^a^	4.99

Means followed by different letters (a–d) are significantly different from the LSD (least significant difference) values at p = 0.05. Significant differences shown are in land uses.

In the 15–30 cm soil layer, land use showed a significant effect on the labile and very labile carbon fractions, whereas the less labile and non-labile fractions were not significantly affected by land use. At this soil layer, very labile carbon, labile carbon, less labile, and non-labile fractions ranged from 2.0 to 5.33 g Kg^–1^ soil, 5.01 to 1.36 g Kg^–1^ soil, 1.31 to 3.16 g Kg^–1^ soil, and 3.98 to 6.90 g Kg^–1^ soil, respectively. The natural forest showed a significantly higher amount of very labile carbon (5.33 g Kg^–1^ soil), followed by the pineapple and fallow *jhum* (2.33 g Kg^–1^ soil), and the lowest was recorded in *jhum* (2.0 g Kg^–1^ soil). A similar trend was also found in the labile carbon, whereas, the less labile and non-labile carbon showed no significant effect. In the 30–45 cm soil layer, labile carbon was found in the highest amount (4.44 g Kg^–1^soil) in the natural forest followed by the pineapple followed by the *jhum* and fallow *jhum* land. However, the *jhum* and fallow jhum are statistically at par with each other. The less labile form of carbon showed a similar trend, while the non-labile form had shown no significant effect; however, the highest amount of non-labile carbon was found in the natural forest land use (4.78 g Kg^–1^ soil), and in the pineapple land-use system.

In the 30–45 cm soil layer, very labile, labile, and less labile fractions showed a significant effect on land use, whereas, the non-labile did not find a significant effect on land use. In this soil layer, very labile carbon, labile carbon, less labile carbon, and non-labile carbon ranged from 1.11 to 2.33 g Kg^–1^ soil, 1.37 to 4.44 g Kg^–1^ soil, 5.45 to 7.75 g Kg^–1^ soil, and 2.87 to 4.78 g Kg^–1^ soil, respectively.

**Figure 2:**
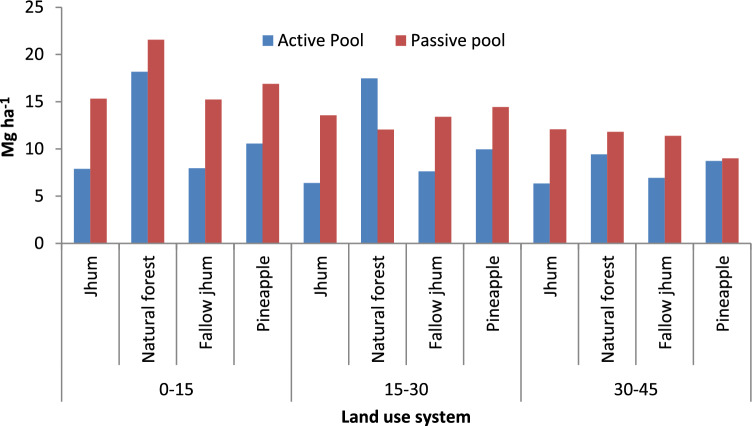
Effect of land use on active carbon (AC) pool and passive carbon (PC) pool ratio).

**Figure 3:**
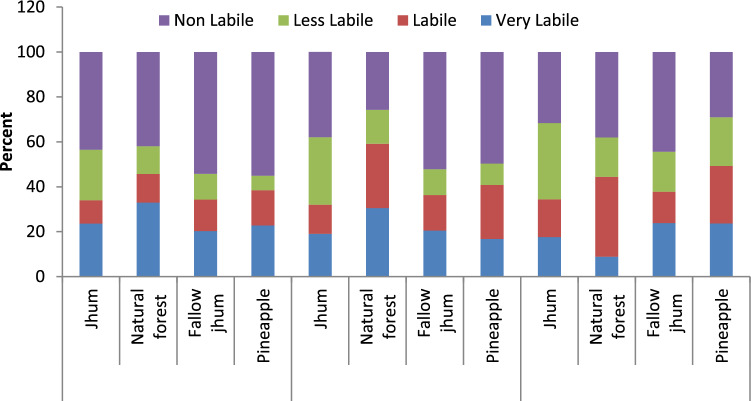
Contribution of different fractions of carbon in total soil organic carbon in different land use.

Across land uses, the active pool in soils ranged from 4.27 to 13.23 Mg ha^−1^ in 0–15 cm, 6.36 to 10.42 Mg ha^−1^ in 15–30 cm, and 3.27– 5.55 Mg ha^−1^ in 30–45 cm depths. Among land-use systems, the natural forest had a significantly higher amount of active pool in different soil layers, followed by pineapple (Fig. 2). The smallest amount of active pool was obtained in soils under *jhum* land-use systems, which were on par with fallow *jhum* land. Similarly, the passive pool was also significantly influenced by the land-use system in the 0–15 cm depth and 15–30 cm soil layer, however, in the 30–45 cm soil layer, it was not significantly influenced by the land-use. It ranged from 7.71 to 11.06 Mg ha^−1^ in 0–15 cm, 7.13 to 8.24 Mg ha^−1^in 15–30 cm and 4.99 to 6.98 Mg ha^-1^ in 30–45 cm soil layers in different land uses. Of the various land-use systems, soils under the natural forest land-use system had the highest passive pools (11.06 Mg ha^−1^) at 0–15 cm, whereas, the lowest passive pool was recorded in pineapple land use at 30–45 cm soil depth. Non labile and less labile carbon pool contributed the most to total carbon in Pineapple and fallow *jhum* land (Fig. 3), indicating that carbon is being rapidly lost from labile pool while nonlabile pool is remaining intact.

### Effects of land use management on soil microbial biomass carbon (SMBC)

The study expressed that the soil microbial biomass carbon (SMBC) and dehydrogenase activities (DHA) were significantly affected by the land use system across the different soil layers. The data divulge that SMBC and DHA were consistently decreasing deeper in the soil layer (Table 5). The surface soil layer of various land uses had the highest SMBC (497–306 μg g^–1^ dry soil). Significantly higher concentrations of SMBC were found in soils under natural forest, followed by pineapple land use, fallow *jhum* and the lowest was recorded in *jhum* land use in the respective soil layers. The highest depletion in SMBC (38.4% at 0–15 cm, 34.9% at 15–30 cm, and 19% at 30–45 cm soil layer) was found in the *jhum*, whereas the lowest depletion in SMBC (14.1% at 0–15 cm, 13.1% at 15–30 cm and 10.8% at 30–45 cm soil layer) was recorded in pineapple and the natural forest. Moreover, the fallow *jhum* showed a little less depletion (29.9% at 0–15 cm, 26.2% at 15–30 and 18.4% at 30–45 cm soil layer) as compared to the *jhum;* this indicates that the fallow *jhum* is in the process of regaining the lost microbial activities.

**Table 5:** Effect of land use change on SMBC and dehyroginase activities.

Land use system	SMBC (µg g^–1^dry soil)			DHA (µg *TPFg^−1^ soil h^−1^)		
	0–15 cm	15–30 cm	30–45 cm	0–15 cm	15–30 cm	30–45 cm
Jhum	306^d^	292^d^	278^b^	43.7^c^	36.3^c^	30^c^
Natural forest	497^a^	449^a^	352^a^	61.7^a^	54.0^a^	50.7^a^
Fallow jhum	348^c^	331^c^	287^b^	44.0^c^	37.7^c^	31^b^
Pine apple	427^b^	390^b^	314^b^	51.7^b^	44.7^b^	32.3^b^

Means followed by different letters (a–d) are significantly different from the LSD (least significant difference) values at p = 0.05. Significant differences shown are in land uses.

*TPF triphenyl formazan.

### Effects of land use management on dehydrogenase activities (DHA)

The highest DHA activity in soils of all land uses was recorded, and it was significantly affected by the different land-use systems. With increasing depth, dehydroginase activity decreased. The highest dehydroginase activity was reported at 0–15 cm depth and the lowest at 30–45 cm depth in all land uses (Table 5). Among land uses, the highest dehydrogenase activities was found in the natural forest land use (61.6 µg TPFg^−1^ soil h^−1^at 0–15 cm, 54 µg TPFg^−1^ soil h^−1^ at 15–30 and 50.7 µg TPFg^−1^ soil h^−1^ at 30–45 cm soil layer) followed by the Pineapple land-use system and lowest DHA was recorded in *jhum* land (43.7 µg TPFg^−1^ soil h^−1^ at 0–15 cm, 36.3 µg TPFg^−1^ soil h^−1^ at 15–30 cm and 30 µg TPFg^−1^ soil h^−1^ at 30–45 cm soil layer). Moreover, the DHA of fallow *jhum* and *jhum* land were found at par at surface soil layer (0–15 cm) and 15–30 cm soil layer, while at the 30-45 cm soil layer, the natural forest is significantly higher and rest all at par.

## Discussion

### Soil pH, bulk density and soil organic carbon

Soils in the natural forest had significantly higher pH, which could be attributed to extensive root systems and more exudates from different vegetation in natural forests affecting soil fertility status^19^, addition of plant residues can increase, decrease or have little effect on soil pH, depending on initial soil pH, The input of plant residues decreases soil pH at high initial soil pH through nitrogen nitrification in the residue. However, at low initial soil pH, the activity of nitrification bacteria will be suppressed and mineralized nitrogen tends to be ammonified and thus increase soil^20^. whereas the jhum-land use system had the lowest pH, followed by Pineapple land use. In the deeper layer at 30–45 cm depth, the effect was less pronounced as some of the leached out bases were expected to be deposited or adsorbed in the lower soil horizons^21^, Due to this phenomenon, lower layers had a relatively higher pH than the surface and subsurface layers, irrespective of land use. Other researcher^22,23^ have also reported variation in soil pH due to different years of *jhum* cultivation and management practices followed in various land-use types. SOC and bulk density had a negative correlation^24,25^. The tendency of bulk density to increase with depth could be attributed to the mass of the overlying soil^26^ and the decrease in SOC^27^ and the fertility of the soil^28^. The natural forest land use showed the lowest bulk density at all the soil depths compared to the other land-use systems in our study, whereas, it was recorded highest in *jhum* and the reverse trend was recorded for SOC. Our findings were consistent with^29^ who found that bulk density and soil organic carbon were negatively correlated in soils of the western Himalayas. However, it is also a well-established fact that bulk density and soil organic carbon are oppositely proportional to each other^30,31^, the higher value of SOC in the soil is an indicator of a lower BD and good aeration.

Changes in land-use systems and subsequent changes in soil organic carbon have strongly affected soil physical properties: most prominently the bulk density and porosity of the soil. The deforestation of natural forests and subsequent burning for shifting cultivation resulted in a significant reduction in soil organic carbon; the reduction of SOC up to a depth of 0–30 cm was significant. The forest had the greatest content of SOC due to its higher net primary productivity, and very high litterfall that covered the soil surface. This litterfall acts like mulch contributing to increasing soil organic matter^32^. As a result, the SOC in the natural forest is high. When this land is converted to shifting cultivation, such as *jhum*, the SOC drastically reduced due to forest burning and oxidation of organic matter^33^. A similar report was also reported by^34^ in the East Siang district of Arunachal Pradesh. This study well explains why deforested land use, viz., *jhum*, fallow *jhum,* and pineapple land use, has undergone depletion of a considerable amount of SOC, and this further reduces the C input due to crop cultivation in the *jhum* land. The fallow *jhum* land-use showing signs of recovery after being fallow for two or more years. As a result, this landuse showed a higher SOC compared to the *jhum* land use. The pineapple land use had a considerable improved SOC over the *jhum* land as it was consistently under pineapple cultivation for the last 7 years (Table 1) after the 2 years of *jhum*.

At large, SOM is presumed to be higher in natural forests than in deforested soils like *jhum* and fallow *jhum* soils due to larger inputs and less intense decomposition of SOM. Contrary to the presented study, The highest concentration of SOM (13.2 g kg^–1^) was found in grassland in the topsoil layer (0–15 cm), followed by forest land (10.2 g kg^–1^) and cultivated (7.23 g kg^–1^) soils^35^. Similar to our study^36,37^ found that conversion of native vegetation to agricultural systems significant impact on SOC, lowering it by 9–25%. The magnitude of the decrease in SOC in their studies was not as large as in our study (41.8–13.4%), probably due to differences in soil type, texture, organic matter source, sampling depth, and *jhumimg* processes and also due to altitudinal locations which influencing SOC controlling temperature regimes, precipitation, solar radiation, relative humidity, and geologic deposition processes^38^. The increasing soil depth decreasing aeration, soil microbial activity, and fine root turnover might have resulted in the corresponding decrease in the concentration of SOC^39^. Our study showed that natural forest systems had a significantly higher concentration of SOC across soil depths, while *jhum* land use had the least SOC.

### Soil organic carbon pools and fractions

Soil carbon pools present in the soil are a product of the equilibrium between carbon addition and carbon depletion^6^. In the present study, the SOC pool was the highest under natural forests, followed by pineapple, whereas, the lowest was found under the *jhum* land-use system. Conversion of carbon-rich natural forests to croplands results in a rapid loss of carbon pool^40^ and threatens the ecosystem’s normal functioning^41^.

The Arunachal Pradesh region of India (the study site) has a cooler climate and receives very high annual rainfall (> 3000 mm per annum). It is expected to have a low rate of mineralization in the soils of these regions owing to high rainfall and a cooler climate compared to the northern Indo-Gangetic plain of India. The active carbon (AC) and passive carbon (PC) pools were significantly affected by the land use system across all depths, except in the 30–45 cm soil layer, where only passive pool land use showed a significant effect with land use. Similar to our study^42^ also reported that land-use changes have significantly impacted the AC and PC pools in soil. The AC pool in the soil acts serves as a source of energy for soil microorganisms and as a predictor of soil quality^16^. Whereas, the PC reserve is not swiftly affected by soil management practices and soil microbial activities^43^. The natural forest had the highest AC and PC pools in 0–15 cm, 15–30 and 15–30 cm depths and *jhum* land use had the lowest AC & PC poll in all three soil layers. In this study, the higher amount of passive C pools in soils under natural forest and Pineapple land-use systems might be due to the continuous addition of C inputs through different types of fine roots, root exudation, forest litters, nominal soil interruption and reduced soil erosion^44^. Similar to our study, which discovered that soil pH influenced the quantitative distribution of soil organic carbon between fractions, more acidic soil is less transformed into an active pool^45^. Statistically similar values of AC pools among the natural forest soil and pineapple land-use systems in 0–15 cm soil depth are mainly attributed due to variation in soil bulk density. The magnitude of variation in active and passive pools is less in deeper soil as compared to the upper soil layer among the different land-use systems.

The amount of carbon reserve depend on the crop biomass productivity that augments the AC fragment^42,46^. The addition of readily decomposable leaf litter throughout the year in the natural forest and pineapple system might have produced a high AC pool. Among the land uses studied, the *jhum* and fallow *jhum* land-use systems had the largest reduction in the AC pool. The *jhum* had deleterious activities while preparing the land for cultivation, which might have increased losses of the AC pool from the soil. Similar variation was also reported by^47^ the variation in AC and PC pools across land uses could be attributed to variations in vegetation type, the addition of litter and the intensity of soil distribution^26^. SignificantlyThe higher very labile carbon fractions in natural forest and pineapple land-use systems compared to fallow land are attributed to the high input of plant litters and protection of added carbon, which increased the microbial activity and thus the very labile carbon fraction. Besides this, the natural forest area was surrounded by *Alnus nepalensis*, which is a non-leguminous N-fixing plant with a higher rate of litterfall. Leaves with better N content have a greater capacity to increase the labile carbon in soil^48,49^. A higher amount of very labile carbon in forest soil was also reported by^50^.

The higher amount of Non-labile carbon (NLC) fractions in soils under natural forest and Pineapple land-use systems in this study could be attributed to minimal soil disturbance, a slow rate of decomposition, and composition of the plant, underlying grasses, and shrubs^51^. Generally, forest litters are rich in tannin and wax, which are resistant to decomposition and may contribute toward greater non-Labile carbon under forest soil than the deforested (*jhum* and fallow *jhum*) land as these materials might be lost during the burning process. However, the higher relative proportion of NLC (32.5 to 37.5%) to SOC in all the depths under deforested land than in natural forest and pineapple land-use systems suggested that losses of the very labile carbon fraction were higher under deforested land due to *jhuming* and soil disturbance as compared to natural forest and pineapple land-use systems. The non-labile carbon fraction is resistant to soil microorganisms decomposition and soil management practises due to sorption on fine particles^43,52^.

### Soil microbial biomass carbon and dehydrogenase activities

In the current study, significant influences of land use on microbial carbon biomass and dehydroginase activities were observed. The natural forest recorded the highest microbial carbon biomass (497 µ g g^–1^dry soil dry soil) followed by pineapple land use, whereas the lowest was reported in the *jhum* and fallow *jhum* on surface soil. The highest microbial carbon biomass was recorded on surface soil and gradually decreases with the inceasing the soil depth. This could be due to forest fire being burn while shifting cultivation. Similar to our findings, reduction in microbial biomass after the fire has been reported in many studies^53,54^. This indicated that the burning killed soil microorganisms, which manifested in the reduction of microbial biomass and microbial activities. Furthermore, burning could also change the nutrient supply and could also be a reason for the reduction in microbial biomass after a forest fire^55,56^. Compared with the land under native forest, the deforested land particularly jhum reduces 38.4% reduction in microbial biomass. The soil microbial biomass content decreased with deforestation, agreeing with the results reported by^57^, who also reported that the soil of degraded land had less microbial biomass than land under native vegetation (undisturbed). The decrease in soil microbial biomass content found in the deforested lands is a consequence of the loss of vegetation cover and SOC from the soil surface. The vegetation covering the soil surface has fundamentally influenced the properties of soils^58^ because it contributes to the input of organic matter to the soil and protects the soil from erosion. Furthermore, the absence of vegetation cover exposes the soil microbial biomass at the soil surface to the direct effects of high temperatures and rain. A similar result was also observed by^59,60^ for African tropical soils and Indian soils, respectively.

The activity of DHA gradually decreases from natural forest to Jhum land use (Table 5) in the order of natural forest > pineapple > fallow Jhum > Jhum. Conversion of natural forest (61.7 µg TPFg^−1^ soil h^−1^) to jhum (43.7 µg TPFg^−1^ soil h^−1^) accounts for a 29.1% loss of DHA. The increase in activity of this enzyme reflects metabolic ability of the soil and its activity is varied with presence of microbial biomass The lower DHA levels imply that the loss of vegetation cover reduced the activity of these enzymes. Similar to aur study it was discovered by^61^ that as vegetation cover decreased, so did the values of enzyme activities in the soils Deforestation decreases the amount of organic matter in the soil that suppresses microbial biomass, thus decreasing the rate of enzymatic reactions^62^. However, the results for soil microbial biomass at the fallow *jhum* land also suggest that the restoration of *jhum* by leaving it for growing vegetation can promote the recovery of soil microbial biomass and DHA. The dynamics responsible for such retrieval could be allowed to regenerate the vegetation that vitalizes the growth of soil microbial biomass by increasing the input of plant roots and plant litter^63^.

## Conclusions

The hypothesis of this study was that transformation of natural forests to deforestation-based land-use systems decreases SOC storage, C fraction pools, and enzymatic activities. While deforested land allows for the regeneration of vegetation and the recovery of the status of SOC, C fraction pools, and enzymatic activities. This study indicates that from natural forest to *jhum* there was a drastic reduction in the SOC storage, enzymatic activities and C fractions, whereas, from *jhum* to fallow *jhum* showed the sign of recovery as all these parameters had improved compared to the jhum land-use system. The fallow phase is a response well to regain the initial soil conditions before the transformation of natural forest to the *jhum* land. The average fallow period allows for the return to initial soil conditions. However, pineapple, the horticulture-based land-use system has further improved all these parameters compared to both deforested land *jhum* and fallow *jhum*. One more important piece of information the non-labile carbon of the pineapple land use was statistically at par with the natural forest. This showedthat when natural forest land is converted into *jhum* or horticulture-based land-use systems, such as pineapple land use, the labile carbon deplete first while the non-labile carbon remain intact.

## Data Availability

The datasets used and/or analysed during the current study are available from the corresponding author upon reasonable request.
